# Landscape determinants of density of blacklegged ticks, vectors of Lyme disease, at the northern edge of their distribution in Canada

**DOI:** 10.1038/s41598-019-50858-x

**Published:** 2019-11-13

**Authors:** Benoit Talbot, Andreea Slatculescu, Charles R. Thickstun, Jules K. Koffi, Patrick A. Leighton, Roman McKay, Manisha A. Kulkarni

**Affiliations:** 1 School of Epidemiology and Public Health, University of Ottawa, Ottawa, ON Canada; 2 Centre for Food-borne, Environmental and Zoonotic Infectious Diseases, Public Health Agency of Canada, Saint-Hyacinthe, QC Canada; 3 Department of Pathology and Microbiology, Faculty of Veterinary Medicine, Université de Montréal, Sainte-Hyacinthe, QC Canada

**Keywords:** Ecological epidemiology, Ecological modelling

## Abstract

In eastern North America, including Canada, Lyme disease is caused by *Borrelia burgdorferi* sensu stricto and transmitted to humans by the blacklegged tick, *Ixodes scapularis*. The last decade has seen a growing incidence of Lyme disease in Canada, following the northward range expansion of *I. scapularis* tick populations from endemic areas in eastern United States. This may be attributable to movement of the many hosts that they parasitize, including songbirds, deer and small mammals. In this study, we wanted to test the effect of spatial, temporal and ecological variables, on blacklegged tick density and infection rates, near the northern limit of their distribution in Ontario and Quebec, Canada. We found an effect of both proportion of forested areas and distance to roads, on density of *I. scapularis* ticks and prevalence of infection by *B. burgdorferi*. We also found an effect of both sampling year and ordinal sampling data on prevalence of infection by *B. burgdorferi*. In six adjacent sites showing evidence of reproducing *I. scapularis* populations, we found that forest composition and structure influenced density of *I. scapularis* ticks. Our results suggest that blacklegged tick density and infection rate in Canada may be influenced by a variety of factors.

## Introduction

Lyme disease is a tick-borne illness caused by several species of *Borrelia* bacteria that are present in parts of Europe, Asia and North America, and transmitted to humans through the bite of a range of *Ixodes* tick species^1^. The disease is highly prevalent in eastern North America, where blacklegged ticks (*Ixodes scapularis*) infected by *Borrelia burgdorferi* sensu stricto may efficiently transmit the infection to humans and domestic animals^1–3^. It is estimated that Lyme disease is responsible for 300,000 cases in the United States each year, accounting for underreporting of the disease^4^. In Canada, Lyme disease incidence has risen dramatically, with the reported number of cases increasing from 144 to 2025 between 2009 and 2017^5^.

Blacklegged tick populations have been increasingly observed in eastern Canada in the past several years, particularly in regions bordering endemic areas in the United States to the south. Their establishment in new areas is attributed to multiple factors, including spatial diffusion from established areas, alongside changes in climate and the environment that have impacted tick survival and the range of blacklegged tick hosts^6–9^. Based on surveillance at 36 sites in eastern Ontario, there is evidence that *I. scapularis* has spread northward at a rate of approximately 50 km/year, in line with previous predictions based on climate change^10,11^. An association between Lyme disease emergence and the advance of blacklegged ticks has been observed in the same region from 2010 to 2016^12^. Although less likely, Lyme disease emergence may also be associated with range expansion of the white-footed mouse, the main reservoir host of *B. burgdorferi* in eastern Canada^13^. The bordering cities of Ottawa in Ontario and Gatineau in Quebec represent a major urban and suburban region situated at the northern range front of blacklegged ticks^13,14^, making this an important region for understanding factors related to tick and tick-borne pathogen emergence.

Northward dispersal of blacklegged ticks may be attributable to movement of the many hosts they parasitize, in particular songbirds, but also deer and other small mammals^1^. Long-distance transport of ticks by the means of migratory songbirds is a major contributing factor for the dispersal of ticks outside of their current range distribution. It has been associated with dispersal of *I. scapularis* and *B. burgdorferi* from the northeastern United States into eastern Canada^15^, and likely also from southern parts of the eastern Canadian provinces, where blacklegged tick populations are now well-established, into the Ottawa-Gatineau region in Canada. Migratory songbirds usually follow flyways that are situated along wetlands and major rivers, through systems known as riparian corridors, during their spring and fall migrations^16–19^. Water bodies of at least 5 hectares are thought to be the most important in these regards^19^. Thus, areas surrounding these landscape elements may be prone to colonization by adventitious populations of blacklegged ticks and *B. burgdorferi*. This may translate to local increases in environmental risk for Lyme disease if suitable hosts exist to support tick populations (i.e. deer) or provide infection to immature ticks (i.e. rodents, other small vertebrates). In contrast, tick movement by the means of mammals, such as deer and small mammals, has been associated with forest fragmentation^20–22^. In Europe, road and freeway edges may facilitate the dispersal of blacklegged ticks as they hitchhike on deer and small mammals in these forest-fringe areas^23,24^. Proportion of canopy cover was found to influence tick density in the United States and Canada^8,25^. Several competing or interacting landscape characteristics may therefore contribute to elevate Lyme disease risk in human populations^26^, through their impacts on local tick abundance^8,23,25^ and *B. burgdorferi* prevalence in ticks^24^.

The identification of landscape features associated with elevated tick abundance and infection rates can help to characterize areas of environmental risk for human Lyme disease infection. This in turn can help to target public health interventions and reduce Lyme disease risk to human populations. This is particularly important in regions of recent and ongoing disease emergence, where risk areas are rapidly changing^6^. In this study, situated at the northernmost limit of the distribution of blacklegged ticks in eastern Ontario and southwestern Quebec, Canada, we assessed variations in the distribution and abundance of *I. scapularis*, and their prevalence of infection with *B. burgdorferi*. We wanted to test the effect of proximity to land cover elements that may be conducive to movement of tick hosts, such as water bodies that are important for bird migration, and roads which fragment the landscape and act as dispersal corridors for mammals, on blacklegged tick density and infection rate across the study area. We considered water bodies of at least 5 hectares because they are the most important for bird migration, and we considered roads of one or two lanes because they are spread out around the study area and are one of the main drivers of landscape fragmentation. We strengthened these analyses with inclusion of time of sampling and proportion of forest cover to account for other processes that may affect tick density and infection rates. Lastly, we investigated the effects of ecological variables related to forest composition and structure on abundance of ticks, at six adjacent sites showing evidence of reproducing tick populations.

## Results

### Study site characteristics and tick density

We collected a total of 576 *I. scapularis* ticks in 33 sites in two rounds of sampling, in late Spring and in early Fall, each year from 2017 to 2018, including 471 adults, 83 nymphs and 22 larvae (Table 1; Supplementary Table S1). A total of 572 were collected from 30 sites in Ottawa (Site O1 to O30; Table 1), while only four ticks were collected from three sites in Gatineau (G1 to G3; Table 1). The majority of ticks were collected from sites in western and south-western areas of Ottawa. Sampling effort varied between 1.5 and 19.2 hours per site (Table 1), depending on meteorological conditions, cluttered understory in certain sites which reduced efficiency of drag sampling, and seasonal closure of certain sites for maintenance.

**Table 1:** Blacklegged tick abundance and infection rate indices around the cities of Ottawa and Gatineau, Canada.

Site ID	Sampling effort	Adult	Nymph	Larva	Total density	Infected adult	Infected nymph	Number tested	Percent infected
O1	14	15	0	0	1	2	0	15	13
O2	7	0	1	0	0	0	0	1	0
O3	12	0	0	0	0	0	0	0	N/A
O4	17	2	0	0	0	0	0	2	0
O5	13	0	0	0	0	0	0	0	N/A
O6	14	1	0	0	0	0	0	1	0
O7	14	32	7	1	3	11	1	39	31
O8	14	0	2	3	0	0	0	2	0
O9	13	61	2	0	5	21	0	63	33
O10	9	25	4	0	3	10	0	29	34
O11	19	27	13	5	2	11	0	40	28
O12	5	12	0	0	2	1	0	12	8
O13	8	20	36	2	7	11	12	56	41
O14	7	0	0	0	0	0	0	0	N/A
O15	12	8	0	0	1	1	0	8	13
O16	12	27	0	11	3	11	0	27	41
O17	7	0	0	0	0	0	0	0	N/A
O18	9	0	0	0	0	0	0	0	N/A
O19	10	7	0	0	1	3	0	7	43
O20	10	137	7	0	14	61	1	144	43
O21	7	19	0	0	3	2	0	19	11
O22	7	2	0	0	0	0	0	2	0
O23	8	50	7	0	7	16	0	57	28
O24	8	4	0	0	1	0	0	4	0
O25	6	0	0	0	0	0	0	0	N/A
O26	6	10	4	0	2	2	1	14	21
O27	2	0	0	0	0	0	0	0	N/A
O28	2	0	0	0	0	0	0	0	N/A
O29	3	8	0	0	3	5	0	8	63
O30	2	0	0	0	0	0	0	0	N/A
G1	3	2	0	0	1	0	0	2	0
G2	3	2	0	0	1	1	0	2	50
G3	3	0	0	0	0	0	0	0	N/A
Total	286	471	83	22	2	169	15	554	33

**Figure 1:**
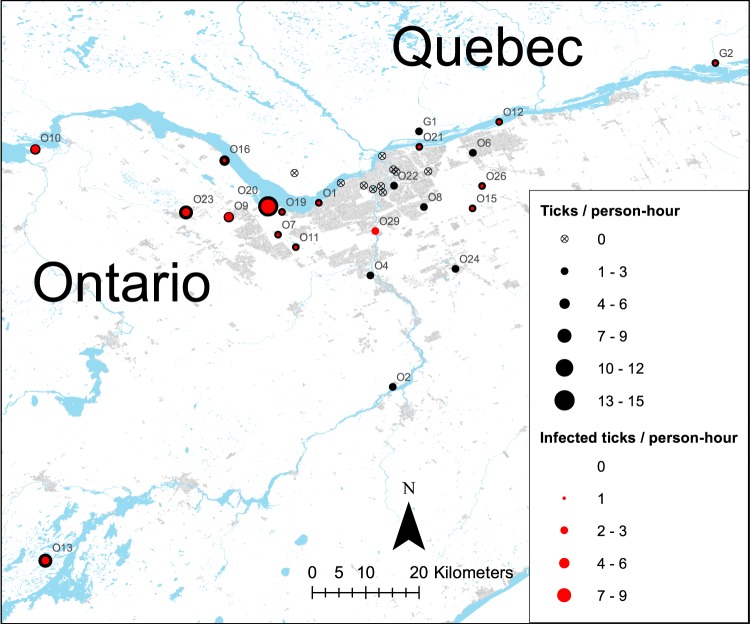
Map of blacklegged tick dragging sites around the cities of Ottawa and Gatineau, Canada. Format, size and shading of points represent number of collected ticks, along with number of ticks infected by *Borrelia burgdorferi*, per hour of sampling, as in Legend. Sites where non-zero tick abundance was observed are labelled by site ID, as listed in Table 1. Built-up land is shown in light grey shading and water bodies in lighter blue shading. Map was created using ArcGIS 10.5 (ESRI, Redlands, CA, United States).

Number of ticks of each life stage, number of adults and nymphs infected with *Borrelia burgdorferi*, and proportion of *B. burgdorferi*-infected ticks out of number tested, collected in 33 sites around the city of Ottawa (site identifiers starting with O) and around the city of Gatineau (site identifiers starting with G; see Fig. 1), in the Spring and Fall seasons of 2017 and 2018, are shown. Sampling effort indicates the total number of person-hours of drag sampling per site; total density indicates the total number of sampled ticks per person-hour.

Straight-line distance to water bodies from study sites’ centroid varied between 6 and 9327 meters (average of 1728 meters), straight-line distance to roads varied between 0 and 386 meters (average of 79 meters), and proportion of forested areas varied between 0 and 85% (average of 29%). Within the six tick-endemic sites in western Ottawa (O7, O9, O11, O16, O20 and O23; Fig. 1), we sampled a total of 241 transects of 100 meters (total of 24.1 km). Nearly three quarters of transects were in areas dominated by maples, cedars or pines; nearly half were in areas with medium abundance of the dominant tree type; more than half were in forested habitat; and nearly three quarters were in areas with flat terrain (Table 2). Most sites were characterized by sparse understory and deep litter layer, although these variables varied considerably among sites (Table 2).

**Table 2:** Variables included in site-specific analysis of factors associated with blacklegged tick abundance.

Predictor	Value	Site identifier						Total
		O7	O9	O11	O16	O20	O23	
Dominant tree type	ash	0	0	4	2	20	0	3
	beech	2	5	16	0	4	7	6
	birch	0	5	4	0	0	0	2
	cedar	80	0	2	39	20	0	24
	elm	0	15	9	0	16	9	7
	fir	0	0	0	2	0	0	0
	maple	11	61	29	2	4	56	29
	oak	2	2	2	0	8	11	4
	pine	2	12	31	44	0	18	19
	poplar	2	0	2	2	8	0	2
	spruce	0	0	0	5	8	0	2
	no tree	0	0	0	2	12	0	2
Abundance of dominant tree type	high	59	34	20	32	12	24	32
	medium	32	56	64	39	0	69	47
	low	9	10	16	24	76	4	19
	negligible	0	0	0	5	12	2	2
Habitat type	forest	68	73	71	44	24	71	61
	woodland	30	22	22	24	24	27	25
	savannah	0	0	2	20	12	2	5
	shrubland	2	5	4	2	40	0	7
	open	0	0	0	10	0	0	2
Understory density	dense	7	7	11	12	32	11	12
	full	30	20	32	50	40	51	37
	sparse	55	73	57	24	28	33	46
	bare	9	0	0	14	0	4	5
Litter layer depth	deep	34	41	47	51	28	36	40
	moderate	5	10	31	22	28	24	20
	shallow	32	44	22	15	44	36	31
	none	30	5	0	12	0	4	9
Site aspect	depression	16	5	13	5	0	9	9
	flat	84	76	78	61	100	38	71
	hilltop	0	10	0	2	0	29	7
	slope	0	10	9	32	0	24	13

Proportion of sampling transects (%) according to categories of predictor variables used in the analysis of variance with repeated measures to explain abundance of ticks collected within six sites in the city of Ottawa in 2018, calculated for each study site separately, and total among all six study sites.

### Tick infection rates

Following qPCR screening assays targeting *B. burgdorferi* DNA in the 554 adult and nymphal *I. scapularis* ticks sampled across the two-year study period, we identified 184 (33%) infected with *B. burgdorferi*, 169 of which were adults, and 15 were nymphs (Table 1). All individuals that tested positive for the 23S rRNA gene also tested positive for the OspA gene. The majority of *B. burgdorferi*-infected ticks were collected from sites from the western and south-western areas of Ottawa, similar to our observed patterns in tick density, with the highest density in O20, a large marshland situated on the southern shore of the Ottawa River (Fig. 1). Almost all infected nymphs were found in O13 (12 out of 15 infected nymphs), a provincial park situated in the southwest of the study area outside of the city of Ottawa (Fig. 1). The overall *B. burgdorferi* infection rate for sites within the city of Ottawa (*n* = 494; Table 1) was 32%, compared to 41% at the provincial park southwest of Ottawa (site O13, *n* = 56), and 25% for sites in Gatineau (sites G1, G2 and G3; *n* = 4), although the latter infection rate should be interpreted with caution owing to limited sample size.

### Factors associated with tick density and infection rates across the study area

Our area-wide analysis, which considered all 33 sites in the study area, attempted to test our hypotheses about proximity to land cover elements potentially conducive to tick host movement, namely water bodies of 5 hectares or more, and roads with one or two lanes, on tick density of all life stages together and separately, and on prevalence of infection by *B. burgdorferi*. Across a total of 22 models constructed for each of the response variables, which included a variety of combinations of predictors, the best-fit model (i.e. model with the lowest AICc) included different combinations of fixed-effects variables, depending on the response variable under scrutiny.

While the best-fit model explaining total number of ticks included both distance to roads and proportion of forested areas, the best-fit model explaining number of adults only included proportion of forested areas. Both models had a high fit (Conditional pseudo-R^2^ = 0.87 and 0.80, respectively; Table 3). The best-fit model explaining number of nymphs and the best-fit model explaining number of larvae both had a relatively low fit (Conditional pseudo-R^2^ = 0.36 and 0.12, respectively; Table 3). They both included proportion of forested areas, although the former also included ordinal sampling date and the latter also included sampling year.

**Table 3:** Model selection results of area-wide analysis of factors associated with blacklegged tick abundance and infection rates.

Model parameters	Total number of ticks	Number of adults	Number of nymphs	Number of larvae	Infection prevalence
Predictors included	3 + 4	3	2 + 3	1 + 3	1 + 2 + 3 + 4
Degrees of freedom	5	4	5	5	7
Log-likelihood	−226.21	−2045.21	−66.19	−31.31	−218.90
AICc	463.12	418.87	143.07	73.31	454.74
Weight	0.25	0.24	0.58	0.28	0.63
σ^2^ for sampling effort	7.7	3.0	3.2	8.1	3.9
σ^2^ for site identifier	2.9	3.9	4.4	6.1	25.2
Marginal pseudo-R^2^	0.08	0.12	0.36	0.12	0.27
Conditional pseudo-R^2^	0.87	0.80	0.36	0.12	0.64

Model selection parameters, model-averaged Akaike weights (Weight), variance (σ^2^) explained by random-effects variables, and marginal and conditional pseudo-R^2^ of the best performing generalized linear mixed-effects model explaining each of five response variables related to blacklegged ticks collected from 33 sites around the cities of Ottawa and Gatineau from 2017 to 2018, from a total of 22 mixed-effects models for each response variable. Predictors included of the models are a combination of Year (1), Ordinal date (2), Proportion of forested areas (3), Distance to roads (4), and Distance to water bodies (5).

Relative variable importance for proportion for distance to roads for predicting total number of ticks was moderately high, at 0.78, and relationship was negative (Table 4), signifying that sites which are farther away from roads were associated with lower density of ticks. Relative variable importance of sampling year for predicting number of larvae was high, at 0.92 and the relationship was negative (Table 4), which means the year 2018 was associated with lower density of larvae than 2017. Relative variable importance of ordinal sampling date for predicting number of nymphs was high, at 1.00, and the relationship was negative (Table 4), signifying that spring season was associated with higher numbers of nymphs.

**Table 4:** Model averaging results of area-wide analysis of factors associated with blacklegged tick abundance and infection rates.

Response variables	Explanatory variables	Estimate	Standard error	RVI
Total number of ticks	Year (2017 to 2018)	−0.21	0.25	0.35
	Ordinal date	−0.09	0.06	0.50
	**Proportion of forested areas**	**0.97**	**0.37**	**0.86**
	**Distance to roads**	**−0.61**	**0.29**	**0.78**
	Distance to water bodies	−0.28	0.38	0.34
Number of adults	Year (2017 to 2018)	−0.14	0.21	0.23
	Ordinal date	0.02	0.06	0.20
	**Proportion of forested areas**	**0.96**	**0.42**	**0.75**
	Distance to roads	0.26	0.29	0.26
	Distance to water bodies	−0.36	0.45	0.27
Number of nymphs	Year (2017 to 2018)	−0.88	0.84	0.31
	**Ordinal date**	**−1.09**	**0.29**	**1.00**
	**Proportion of forested areas**	**2.51**	**0.67**	**1.00**
	Distance to roads	−0.26	0.38	0.27
	Distance to water bodies	−0.48	0.42	0.31
Number of larvae	**Year (2017 to 2018)**	**−2.84**	**1.16**	**0.92**
	Ordinal date	0.65	0.87	0.24
	Proportion of forested areas	1.52	0.99	0.44
	Distance to roads	−1.79	2.54	0.27
	Distance to water boudies	−0.36	0.87	0.20
Infection prevalence	**Year (2017 to 2018)**	**−1.20**	**0.31**	**1.00**
	**Ordinal date**	**0.28**	**0.05**	**1.00**
	**Proportion of forested areas**	**1.67**	**1.11**	**0.77**
	**Distance to roads**	**2.81**	**0.68**	**1.00**
	Distance to water bodies	0.39	1.13	0.37

Model-averaged coefficients of five predictor variables explaining five response variables related to blacklegged ticks collected from 33 sites around the cities of Ottawa and Gatineau from 2017 to 2018, from a total of 22 mixed-effects models for each response variable. Bold rows correspond to variables with relative variable importance (RVI) > 0.6.

The best-fit model explaining prevalence of infection by *B. burgdorferi* included sampling year, ordinal sampling date, proportion of forested areas, and distance to roads, with a relatively high model fit (Conditional pseudo-R^2^ = 0.64; Table 3). Relative variable importance of sampling year, ordinal sampling date and distance to roads were high, at 1.00 for all three variables (Table 4). Relationship was negative for year of sampling, signifying year 2018 held lower infection prevalence than 2017. Relationship was positive for ordinal sampling date and distance to roads, signifying that ticks collected at a later sampling date or in a site that is situated farther away from roads yielded higher infection prevalence. Relative variable importance of proportion of forested areas was moderately high, at 0.77, and relationship was positive, meaning sites that are surrounded by larger forest cover are associated with higher infection prevalence (Table 4).

Proportion of forested areas and distance to water bodies showed a correlation coefficient of *r = *0.4. However, all other correlation coefficients between pairs of predictors were low among all numerical predictors (*r* < ±0.1), and Moran’s I calculated with the full model incorporating all predictors was non-significant (*P* > 0.2). We kept all predictors in our analyses due to low probability of any major collinearity or autocorrelation effect that could affect the outcomes of our analyses.

### Factors associated with tick abundance at specific sites

Our site-specific analysis, which considered six sites in the western end of the city of Ottawa with established tick populations (O7, O9, O10, O11, O16 and O23; Table 1; Fig. 1), tested the effect of ecological variables on tick abundance of all life stages and of the whole tick population.

**Figure 2:**
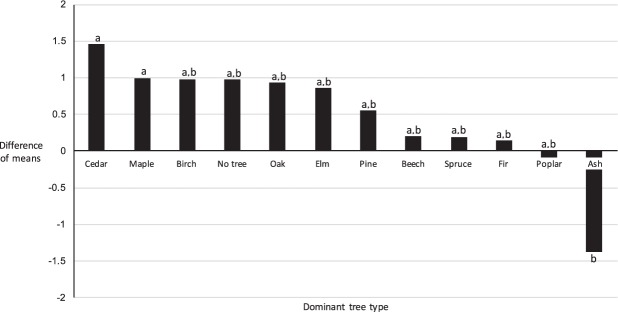
Histogram of difference of means for each dominant tree type on number of blacklegged ticks. Data was collected through tick dragging of 100-meter transects in six sites in Ottawa in 2018 (O7, O9, O10, O11, O16 and O23; Table 1; Fig. 1). Letters next to bars represent significantly different (*P* < 0.05) groups of means of dominant tree types.

We found a significant effect of dominant tree type on total number of ticks and on number of adults (Table 5). In the analysis of total number of ticks, the difference of means was significantly higher in locations characterized by cedar and maple trees, but without ash trees, than vice versa (Fig. 2). This effect was also present in the analysis of abundance of adult ticks, except for maple trees, which did not have any effect on the difference of means. These results suggest that transects with cedars or maples as dominant tree types are associated with higher numbers of ticks, while transects dominated by ash trees are associated with lower numbers of ticks.

**Table 5:** Analysis of variance results of site-specific analysis of factors associated with blacklegged tick abundance.

Response variables	Explanatory variables	Degrees of freedom	Sum of squares	*P*
Total number of ticks	**Dominant tree**	**11**	**72.0**	**0.002**
	Abundance of dominant tree	3	4.6	0.580
	Habitat type	4	19.4	0.084
	Understory density	4	3.0	0.861
	Litter layer depth	3	7.3	0.375
	Soil moisture	3	1.3	0.907
	Site aspect	3	3.4	0.693
Number of adults	**Dominant tree**	**11**	**65.5**	**0.003**
	Abundance of dominant tree	3	3.7	0.643
	**Habitat type**	**4**	**24.3**	**0.030**
	Understory density	4	2.0	0.924
	Litter layer depth	3	6.5	0.407
	Soil moisture	3	1.0	0.933
	Site aspect	3	2.5	0.775
Number of nymphs	Dominant tree	11	1.6	0.202
	Abundance of dominant tree	3	0.1	0.718
	Habitat type	4	0.4	0.442
	Understory density	4	1.0	0.069
	Litter layer depth	3	0.3	0.388
	Soil moisture	3	0.2	0.679
	Site aspect	3	0.2	0.581
Number of larvae	Dominant tree	11	0.1	0.316
	Abundance of dominant tree	3	<0.1	0.289
	Habitat type	4	<0.1	0.774
	Understory density	4	<0.1	0.941
	Litter layer depth	3	<0.1	0.442
	Soil moisture	3	<0.1	0.528
	Site aspect	3	<0.1	0.889

Coefficients from analyses of variance with repeated measures of seven predictor variables explaining four response variables related to blacklegged ticks collected from six sites within the city of Ottawa in 2018. Bold rows correspond to variables with *P* < 0.05.

**Figure 3:**
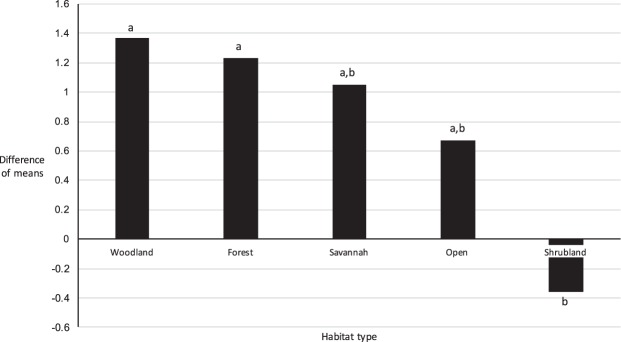
Histogram of difference of means for each habitat type on number of adult blacklegged ticks. Data was collected through tick dragging of 100-meter transects in six sites in Ottawa in 2018 (O7, O9, O10, O11, O16 and O23; Table 1; Fig. 1). Letters next to bars represent significantly different (*P* < 0.05) groups of means of habitat types.

We also found a significant effect of habitat type on number of adult ticks (Table 5). The difference of means was higher in locations characterized by forest and woodland (canopy cover of 50% and higher), and without shrubland, than vice versa (Fig. 3). This suggests that tick abundance in environments with high canopy cover is significantly higher than tick abundance in shrubland environments, in our study area.

Generalized variance inflation factors calculated for each predictor in a full generalized linear model showed values below 2 for all predictors. We kept all predictors in our analyses due to low probability of any major collinearity effect that could affect the outcomes of our analyses.

## Discussion

Previous studies suggest that migratory songbirds may be an important means of long-distance dispersal for ticks^15^, and that songbirds usually follow riparian corridors to reach their wintering or reproduction grounds through major flyways used by multiple species^18,19^. Adult ticks may colonize new areas outside of their native range, and possibly establish founding populations if climate conditions, host density and habitat are appropriate for tick survival and reproduction^11,13,14^. Our results do not support that proximity to water bodies of at least 5 hectares may be associated with higher likelihood of tick establishment and environmental risk for Lyme disease in the Ottawa-Gatineau region. However, additional data on abundance at migratory stopover points and most likely migration routes of various species of migratory birds are needed to clarify the influence of water bodies on tick population establishment. Our results nonetheless suggest that establishment of adventitious tick populations may not necessarily be happening in the vicinity of migratory dispersal corridors, and potentially may require additional landscape features to be successful.

Landscape fragmentation has been linked with increased blacklegged tick density and higher Lyme disease incidence^20,26^. Among others, roads may facilitate movement of ticks through hitchhiking on their mammalian hosts, as seen in two studies in Europe^23,24^. As per our predictions, we found a negative effect of distance to roads of one or two lanes on the density of the whole blacklegged tick population. Therefore, our results support that proximity to roads of one or two lanes may be associated with higher likelihood of tick establishment. However, opposite to our predictions, we detected a positive relationship between distance to roads and infection prevalence by *B. burgdorferi* in blacklegged ticks. A complex interplay between forest fragmentation and diversity of blacklegged tick hosts, with variable competency for *B. burgdorferi*, may be influencing pathogen transmission rates^20,26^, and may not be fully captured by our analysis.

Our study detected inter-annual differences in the density of larval ticks and infection rates of adult and nymphal ticks in our study area, after considering differences in sampling effort between years. We found a higher densityof larvae and nymphal and adult infection rates in 2017 than in 2018. With no other information, it is impossible to ascertain the exact cause of these observed differences. Cumulative growing degree days, or total degrees above 10 °C since the beginning of the year, calculated using daily mean temperatures, through week 20 of the year, was shown to be the best predictor of the start week of the Lyme disease season in northeastern United States^27^. Climatic and weather patterns, including drought, may also affect development, survival and host-seeking behavior of blacklegged ticks^28^. While this investigation was outside the scope of the current study, it is possible that variation in meteorological patterns across years may affect the start of the reproductive activity in blacklegged ticks and may affect abundance and density of host-seeking blacklegged ticks throughout the warm season. Analysis of climatic and weather patterns and tick abundance data over multiple years may help to better understand the drivers of inter-annual fluctuations in this region.

Our results suggest seasonal variation in tick abundance and the abundance of ticks infected with *B. burgdorferi*. We found a higher abundance of nymphs early in the year, as expected based on tick phenology^29,30^. Interestingly, we also found a higher infection prevalence by *B. burgdorferi* late in the year, which is similar to findings from another study in Europe^31^, although this trend is not consistently observed in all regions or all years^32^. Our results support a general pattern seen throughout the range of *I. scapularis*, with the Spring and Fall seasons posing different levels of Lyme disease risk owing to changes in environmental risk and human exposure. The late Spring season brings increased human exposure during outdoor activities in addition to larger numbers of nymphal *I. scapularis*, which are harder to detect and promptly remove than adult ticks, and therefore pose the highest risk of Lyme disease transmission to humans^29,33^. On the other hand, the Fall season may be characterized by more *B. burgdorferi*-infected ticks in the environment, but lower human-tick exposure and the predominance of adult ticks during this season reduces the overall risk of Lyme disease transmission to humans. Further data collection over multiple sampling periods and sites is needed to better understand the factors that may contribute to the observed differences in infection prevalence in the Spring and Fall seasons.

We found a positive relationship between proportion of forested areas and several indices of tick establishment or Lyme disease environmental risk across the study area: total abundance of ticks, number of adult ticks, number of nymphs and infection prevalence by *B. burgdorferi*. Additionally, in our site-specific analysis, areas with tree/shrub cover >50% were associated with higher abundance of adult ticks than shrublands, with tree cover around 0%. This is in contrast to a study performed at a large spatial scale across all of southern and eastern Ontario, which found no such association with tick abundance^11^. On the other hand, a study at a relatively small spatial scale in eastern Ontario found an effect of canopy cover on nymphal blacklegged tick abundance^25^. Another study at a relatively small spatial scale in Virginia found forest cover to be the most important driver of *I. scapularis* density^8^. Altogether, spatial scale appears to be important in determining the effect of ecological variables on tick abundance, and forest cover could be an important determinant of tick abundance within a specific site.

Finally, our results show that transects dominated by cedars and maples held the highest abundance of *I. scapularis* ticks, while locations dominated by ash trees held significantly lower tick numbers. These results may be explained, at least in part, by the behavior of the preferred reproductive hosts of blacklegged ticks, namely white-tailed deer^1^. A previous study in eastern Ontario found that the abundance of white-tailed deer was positively correlated with nymphal blacklegged tick abundance^25^. Similarly, forest stands used by roe deer in Europe was associated with *Ixodes ricinus* abundance^34^. Studies in the Midwest of the United States have shown deer herbivory to be strongest on northern white cedars, eastern hemlocks and white pines^35,36^. Deer are known to follow forest edges of certain conifer species, including balsam firs in Anticosti Island in eastern Quebec^37^ and northern white cedars in northern Michigan^38^, during winter to minimize locomotion costs and increasing access to forage during the cold season. Therefore, if deer aggregate around certain conifer species in the Spring and Fall seasons around Ottawa, these trees would be associated with higher tick abundance in the following warm season. Additionally, maple forests have frequently been associated with higher rates of *I. scapularis* survival in southern Ontario^39^. Another possible explanation for these results is about the difficulty of sampling ticks across forests dominated by certain tree species, compared to other tree assemblages, due to clutter at the understory level, which restricts sampler movement. One limitation of our study is the variation in sampling effort across sites, which can lead to significant differences in tick densities at the local scale^40^.

In conclusion, our study presents a contemporary picture of the distribution of *I. scapularis* ticks and their prevalence of infection with *B. burgdorferi* in a zone of recent and ongoing Lyme disease emergence in eastern Canada^12,41^. Using data from active tick surveillance over two consecutive years, we identified factors that are associated with higher tick density. We also identified spatial and temporal variation in tick infection rates, with an estimated 33% of blacklegged ticks infected with *B. burgdorferi* over this time period. These results can help to characterize variation in environmental risk for Lyme disease in this densely populated region, and identify potential entry points for intervention to reduce human-tick exposure. These effects should be investigated in other emerging blacklegged tick-prone areas across Canada. Among others, the effects of distance to roads on infection prevalence, the increase in infection prevalence late in the year, the overall inter-annual variation in density and infection rates of ticks, and the effects of forest composition on tick density are surprising, and will need further investigations. Finally, more research is needed to better understand how landscape features interact with wildlife host and tick populations through time in this region to facilitate tick establishment and Lyme disease transmission, in order to better predict areas that may be at highest risk of Lyme disease establishment and spread.

## Methods

### Study location

Our study area encompassed 30 sites within and bordering the city of Ottawa (ON, Canada) and three sites within and bordering the city of Gatineau (QC, Canada), which are separated by the Ottawa River. The Ottawa-Gatineau region is comprised of a highly built-up downtown core, surrounded by large suburban and rural areas, as well as actively managed forests and conservation areas. To assess the abundance of blacklegged ticks in a variety of environments across the study area with potential risk of human exposure to ticks, we selected parks, recreational trails and conservation areas that were spatially representative of the study region as our study sites (Fig. 1). We did not select sampling sites based on prior knowledge about local blacklegged tick densities, to avoid sampling bias which could affect the outcomes of our analyses.

### Field data

A team of two to three trained field collectors visited each site in the Spring (May-July) and Fall (September-November) seasons of 2017 and 2018. In each site, ticks were collected for a minimum of 1.5 person-hours (i.e. combined number of sampling hours for all collectors present on any given day), using the drag sampling method, which consists of dragging a white flannel sheet along the ground to collect questing ticks^42^. Our sampling effort varied based on meteorological conditions, cluttered understory in certain sites which reduced efficiency of drag sampling, and seasonal closure of certain sites for maintenance. Collectors stopped roughly every 50 meters to remove ticks from the flannel and collect GPS coordinates. We preserved all live-collected ticks in small plastic tubes and stored them at −20 °C upon arrival at the laboratory.

During the second year of sampling (i.e. 2018), we selected a subset of sites in the western end of the city of Ottawa, where tick abundance and infection rates where highest, to collect additional site-specific data, which were derived from several studies^25,40,43^, for an additional, complementary analysis. These sites included those where we consistently collected ticks of multiple life stages across sampling seasons in 2017, thus reflecting sites with potential reproducing populations of *I. scapularis*^44^. Selecting sites with reproducing tick populations was likely to capture signals from local ecological drivers of tick density. In a total of six sites (O7, O9, O10, O11, O16 and O23; Table 1; Fig. 1) covering an area of about 150 square kilometers, we visually estimated field data for seven variables. We identified the dominant tree type in the area (maple, elm, poplar, oak, beech, birch, ash, pine, spruce, fir, cedar, or no tree; using the taxonomic key from Bertrand *et al*.^45^), the relative abundance of the dominant tree (high: >75%, moderate: 25–75%, low: <25%, or negligible: ~0%), the habitat type (bare: tree/shrub cover ~0%, shrubland: shrub cover >25% and tree cover ~0%, savannah: tree/shrub cover between 25–50%, woodland: tree/shrub cover between 50–75%, or forest: tree/shrub cover >75%), understory density (open: <25%, sparse: 25–50%, full: 50–75%, dense: >75%), litter layer depth (shallow: 0–2 cm, moderate: 3–5 cm, or deep: >5 cm), soil moisture (dry, fresh, moist or wet) and site aspect (flat, depression, slope or hilltop). We collected site-specific field data midway between every two 50-meter sampling points in the course of tick dragging, and linked these data to the number of collected *I. scapularis* ticks of each life stage totaled for the corresponding two locations. This resulted in 100-meter transects as the measurement unit for each variable. This was done over two rounds of sampling per site, one in late Spring and one in early Fall, and over a total of 6 person-hours per site, i.e. 3 person-hours per round of sampling per site.

### Spatial data

We derived the centroid coordinates for each site using ArcGIS v10.5 (Redlands, CA, United States). We obtained spatial land cover data of water bodies (rivers, lakes and ponds), roads (local and arterial roads) and forest patches, from the CanVec Series topographic datasets produced by Natural Resources Canada^46^. We calculated the shortest straight-line metric distance from each site’s centroid to the nearest water body and to the nearest road, using ‘Spatial Analyst’ in ArcGIS v10.5. We drew concentric buffer zones of an area of 1 km^2^ at each study site’s centroid, and calculated the intersecting area between each site’s buffer zone and the forest patch layer, using ‘Spatial Analyst’ in ArcGIS v10.5. For mapping purposes, we calculated tick density as the total number of ticks divided by the person-hours of sampling effort.

### Laboratory analyses

For all collected ticks, we identified the species and stage using microscopy and taxonomic keys^47^. We tested *I. scapularis* adult and nymphal ticks for *B. burgdorferi* using quantitative polymerase chain reaction (qPCR) assays according to previously published protocols^48–50^. Briefly, we extracted total genomic DNA using the QIAamp DNA mini kit (QIAGEN Inc., Mississauga, ON, Canada) and we used qPCR screening assays targeting 23S rRNA to identify presence of *Borrelia* DNA. For positive samples, we conducted a second qPCR assay targeting the *OspA* gene to confirm presence of *B. burgdorferi* DNA.

### Statistical analyses

We conducted two sets of analyses to assess (1) factors associated with tick density and infection rates across the study area at all 33 sites, and (2) factors associated with tick abundance at six tick-endemic sites.

For the area-wide analysis, we used a mixed-effects modeling approach to determine the effect of year of sampling (2017 and 2018), ordinal date of sampling, proportion of forested lands, distance to nearest water body of 5 hectares or more and distance to nearest road of one or two lanes on five response variables: total number of ticks, number of adults, number of nymphs, number of larvae, and prevalence of *B. burgdorferi* infection in adults and nymphs (in percentage, without decimals). We used the ‘glmer’ function from the ‘lme4’ package^51^ in R 3.5.1. We performed the analysis on every possible combination of fixed-effects variables, including a null model excluding all fixed-effects variables. We included site identifier (i.e. to account for repeated sampling in a number of sites) and sampling effort (i.e. to account for varying number of person-hours of sampling across sites) as random-effects variables. We assessed variable importance using model averaging^52^. We used the Poisson modeling family, due to the zero-inflated positive nature of the response variables. We calculated a marginal and conditional pseudo-R^2^ for each model, following the method of Johnson^53^, using the ‘r.squaredGLMM’ function from the ‘MuMIn’ package^54^ in R 3.5.1 to measure the strength of the effect of predictors in our models.

Prior to these analyses, we tested for correlation among pairs of predictors, to detect potential collinearity effects on the results. We also tested for spatial autocorrelation of data points, with the ‘Moran.I’ function in the ‘ape’ package^55^ and a matrix of inverse spatial distances as ‘weights’, in R 3.5.1. Finally, we standardized all numeric variables, by subtracting the mean and dividing by the standard deviation, for more meaningful comparisons among predictors and to remove dispersion issues in the models.

For the site-specific analysis, we conducted an analysis of variance with repeated measures to determine the effect of dominant tree type, abundance of dominant tree type, habitat type, understory density, litter layer depth, soil moisture and site aspect on four response variables: total number of ticks, number of adults, number of nymphs, and number of larvae. To this end, we used the ‘aov’ function from the ‘stats’ package in R 3.5.1. We used the site identifier as error term to correct for repeated sampling at each site, which could bias our analysis due to pseudo-replication. For variables displaying significant effect (*P* < 0.05), we conducted a post hoc test to determine which levels in the variables display a significant difference in means. To this end, we used the ‘TukeyC’ function from the ‘TukeyC’ package^56^ in R 3.5.1.

Prior to these analyses, we calculated generalized variance inflation factors adjusted for the number of degrees of freedom for all predictors in a generalized linear model, to detect potential collinearity effects on the results. We used the ‘glm’ function from the ‘lme4’ package^51^ and the ‘vif’ function from the ‘car’ package^57^ in R 3.5.1.

## Supplementary information

Supplementary Table S1

## Data Availability

All area-wide data can be found in the Supplementary Table S1.
